# Incidence and seasonality of respiratory syncytial virus hospitalisations in young children in Denmark, 2010 to 2015

**DOI:** 10.2807/1560-7917.ES.2018.23.3.17-00163

**Published:** 2018-01-18

**Authors:** Martin T. Jepsen, Ramona Trebbien, Hanne Dorthe Emborg, Tyra G. Krause, Kristian Schønning, Marianne Voldstedlund, Jens Nielsen, Thea K. Fischer

**Affiliations:** 1 Statens Serum Institut, Copenhagen S, Denmark; 2 These authors contributed equally to this manuscript.; 3 Department of Clinical Microbiology, Hvidovre University Hospital, Hvidovre, Denmark; 4 Department of Clinical Medicine, Faculty of Health and Medical Sciences, University of Copenhagen, Denmark.; 5 Department of Infectious Diseases and Centre of Global Health, University of Southern Denmark, Odense, Denmark

**Keywords:** Respiratory syncytial virus, RSV, disease burden, cost-benefit, incidence

## Abstract

For future decisions on respiratory syncytial virus (RSV)-vaccination strategies and implementation into national immunisation-programmes, we used national registry data (hospitalisation, microbiology and vital statistics) to determine the age-specific incidence and direct medical costs of annual RSV-associated admissions in children < 5 years-old for the period of 2010–2015. We identified ca 2,500 RSV-associated hospitalisations annually amounting to total direct medical-costs of ca EUR 4.1 million per year. The incidence of RSV-associated hospitalisations peaked in infants 1–2 months of age followed by infants 2–3 months of age, and infants < 1 month of age, respectively. Infant boys were at higher risk of severe RSV infection as compared to infant girls: male-to-female ratio peaked with 1.4 at four months of age and gradually levelled out with increasing age to 1.0 at 4 years of age. Five RSV-associated deaths were identified. Our findings demonstrate that in a western country as Denmark, RSV constitutes a considerable burden on childhood health. Furthermore, the best approach to reduce the high incidence of RSV-associated hospitalisations in young infants < 3 months of age may be maternal vaccination due to general challenges in achieving sufficient and protective immune responses in young infants.

## Introduction

Respiratory syncytial virus (RSV) is the most common cause of hospitalisation among children admitted for obstructive airway disease throughout the world [1,2]. Although RSV infections occur frequently in all age groups the youngest infants have the highest rates of RSV respiratory disease [3-7]. Globally, an estimated 3.4 million children younger than 5 years of age are hospitalised each year with severe RSV lower respiratory tract infection (LRTI), with the highest incidence in children younger than 6 months of age [1]. Studies have demonstrated a peak incidence of LRTI occurring between 2 and 4 months of age [8,9]. RSV infection typically manifests clinically with cough, fever and nasal congestion in combination with a characteristic wheezing [10] which is caused by inflammation (bronchiolitis) and blockage of the narrow airways. Young age, prematurity, heart and lung diseases and/or immunosuppression are risk-factors for severe outcomes of RSV infection [11]. Natural RSV infection does not confer long lasting immunity against subsequent infection, and consequently, RSV infections may reoccur throughout a lifetime [11-13].

RSV is the only agent of the three most common organisms that cause death from respiratory-tract infections in infants (RSV, *Streptococcus pneumoniae*, and *Haemophilus influenzae*)*,* for which no vaccine is currently available [14]. Prophylactic treatment with RSV-specific neutralising antibodies often is the only preventive as well as therapeutic option left for high-risk infants in privileged resource settings [15]. Prevention of severe RSV disease through active immunisation of infants and/or during pregnancy would be optimal, but this has in previous immunisation attempts been extremely challenging [16]. Currently, multiple RSV vaccine candidates are undergoing development for human use, and some vaccines for infant use are in phase 3 of clinical testing [17,18]. The vaccine types include recombinant live-attenuated vaccines as well as a variety of inactivated candidates, either for vaccinating the infant shortly after birth or vaccinating the mother during pregnancy to boost maternal antibodies and thus confer protection of the child [19]. In preparation for licensure of vaccines, it is pertinent to update disease burden estimates to ensure a solid evidence-based platform for decisionmaking with regards to implementation of future RSV vaccines. 

In 2015, the World Health Organization (WHO) has made it a high priority to establish globally compatible RSV disease burden surveillance systems and robust age-specific estimates are particularly in focus in order to target optimal age for immunisation [20]. A considerable fraction of RSV disease burden studies are based on data collected at sub-regional or single-hospital level or data collected as a part of vaccine trials [7,21,22]. Larger national as well as international disease burden studies are often based on estimates of the RSV burden from acute LRTIs’ surveillance and laboratory results [23]. 

In this population-based study, we took advantage of the unique national registries available in Denmark. We linked information on hospital admitted individuals registered with an RSV diagnosis in the national patient registry (NPR) with information on RSV testing using a national microbiology test database (MiBa) [24]. We estimated age-specific RSV hospitalisation rates in children below 5 years of age in the period from 1 January 2010 to 26 June 2015. In addition, we described seasonality and trends over time, the average length of hospital stay and the direct healthcare-related costs associated with severe RSV disease.

## Methods

### Study design and setting

We performed a retrospective population-based study of all children younger than 5 years of age and born in or immigrated to Denmark in the period from 1 January 2010 to 26 June 2015 to determine age- and sex-specific rates and trends of RSV-associated hospitalisations. Data on national RSV-associated hospitalisations were linked to data on RSV test results obtained from the MiBa [24].

Denmark has a temperate climate, occupies an area of 42,895 km^2^, and has a population of 5,647,923 as of January 2016. The yearly birth-cohort in Denmark is ca 60,000 individuals and the number of children below 5 years of age in the study period 1 January 2010 to 26 June 2015 was 315,883. Healthcare is free of charge in Denmark, and no private alternative is available for hospitalisation of children with respiratory illness. All microbiology laboratories testing human samples are public and an electronic copy of all test reports are transferred to MiBa. Over the study period, there was no major change in RSV testing practices and the prevalence of RSV-positive tests remained relatively constant during this time (data not shown).

### Registries

The Danish Civil Registration System (CRS) was established on 1 April 1968 and since then a unique personal identification number has been assigned to all Danish residents. CRS contains continuously updated information on vital status. Census data were retrieved from Statistics Denmark, StatBank Denmark from 1 January 2010 to 30 June 2015.

MiBa includes all microbiological test results from the 11 departments of clinical microbiology in Denmark since 1 January 2010. The records include information on unique personal identification number, sample date, laboratory analysis, test result and which laboratory performed the analysis [24].

The Danish NPR contains information on all hospitalisations in Denmark since January 1977, including outpatient treatments since 1995. Individual information on dates of admission and discharge, and diagnoses are registered for every hospitalisation. Since 1994 diagnoses are classified according to the World Health ICD-10 (*International Statistical Classification of Diseases and Health-related Problems*, *10th Revision*) [25]. To identify acute LRTI-associated hospitalisations including RSV-associated hospitalisations, we extracted the ICD-10 diagnoses as listed in Table 1.

**Table 1:** ICD-10 code used for identification of respiratory syncytial virus (RSV) associated hospitalisations for the entire study period, Denmark, 1 January 2010–26 June 2015 (n = 12,330 hospitalisations)

Diagnostic category	ICD-10 code	Number	Percentage
**RSV specified^a^**			
RSV as the cause of diseases classified to other chapters	B97.4	916	7.4
RSV pneumonia	J12.1	3,844	31.2
Acute bronchitis and acute bronchitis with bronchospasm	J20.5	3,517	28.5
Acute bronchiolitis	J21.0	2,566	20.8
**Total RSV specified^a^**	**NA**	**10,843**	**87.1**
**RSV ‘other’^a^**	**^b^**	**1,487**	**12.9**
Total	NA	12,330	100

MiBa: Danish National Microbiology Database; NA: not applicable.

^a^ The ICD-10 codes are divided in (i) RSV specified and (ii) other respiratory diagnoses (RSV ‘other’), where a positive RSV diagnostic test result was identified in MiBa, respectively.

^b^ Influenza: J09.X, J10.X, J11.X; pneumonia, non-influenza viruses: J12.X, J16.X, J17.X, J18.X; pneumonia, virus unspecified; J18.9; bronchitis and bronchiolitis: J20.X, J21.X, J40.X, J42.X; not further specified lower respiratory infection: J22.X. X indicates all diagnoses starting with this code.

The Danish Register of Causes of Death [26] was used to identify RSV-coded deaths and data were available from the period of 1 January 2010 to 31 December 2014. There were no records for 2015.

### RSV case definition

A RSV case was defined as a child younger than 60 months of age (i.e. < 5 years-old), who had been registered as an inpatient in the NPR, that is, hospitalised, with an RSV-specific diagnosis or admitted to hospital with another respiratory diagnosis, but with a laboratory-confirmed positive RSV test result within a period of +/− 7 days spanning the hospital admission. If several RSV-associated hospitalisations were recorded for an individual, only episodes > 30 days apart were included as a separate RSV hospitalisation. This interval was considered adequate in length to distinguish episodes of RSV-associated hospitalisations, as only few cases would be expected to have more than one episode of RSV-associated hospitalisation. Thus, visits to the emergency departments have not been included in the study if they have not resulted in hospitalisation. Paediatric intensive care unit (ICU) data were also not taken into account.

### RSV-associated mortality

Two methods were employed to identify RSV-associated deaths. Any death caused by RSV and registered in the Register of Causes of Death was the direct method. The other indirect method captured deaths in individuals who had died either during hospitalisation or within 30 days after hospital discharge with an RSV-specific diagnosis and/or positive RSV test result linking vital status in the CRS and hospitalisation in the NPR. Deaths were reviewed on a case basis including information (ICD-10 codes) on comorbidity and considered RSV-associated, unless another likely cause of death was included in the most recent ICD-10 codes associated to the deceased before death.

### RSV seasons

RSV seasons were analysed on an annual basis using a calendar year starting in week 33 during the last warm summer month of August with median temperatures of 17 °C and ending in week 32 the following year, which was in the middle of the period when fewest RSV cases were observed. For the first season 2010, only RSV cases admitted to the hospital from 1 January 2010 to week 32 in 2010 were available.

### Direct healthcare expenses associated with RSV-hospitalisations

The length of hospitalisation was calculated from admission date to discharge date from the NPR. The algorithm used to define courses of admissions has previously been described [27].

The expenses related to the public healthcare system are centrally regulated and according to the Danish Health Data Authority, the price per hospitalisation for RSV-associated disease < 6 days is EUR 2,000 (Danish kroner (DKK) 14,892 at a May 2016 exchange rate of DKK 744 to EUR 100) [28]. If the hospitalisation is 6 days or longer, the cost is EUR 262 (DKK 1,951) per 24 hours of added stay. These costs are total costs and based on average expenditures for mild as well as severe RSV-associated hospitalisations in Denmark. The costs include all hospitalisation-associated expenditures (e.g. hospital bed, diagnostic work up, healthcare personnel, rehydration treatment and intensive care) and cannot be segregated further into various expenditure categories.

### Statistical analyses

The numbers of children younger than 5 years who were hospitalised for RSV-associated disease in Denmark were calculated according to age groups: incidence rates were estimated per 1,000 in the following age groups; < 1 month, 1, 2, 3, 4, 5, 6, 7, 8, 9, 10, 11 months, 0–11 months, 12–23 months, 24–35 months, 36–47 months and 48–59 months. Patients with a positive laboratory test, but without a registered hospitalisation, were excluded as these cases were not considered severe. Pvalue for RSV incidence was calculated using the chi-squared test. All descriptive and statistical analyses were carried out in Stata version 12.1.

## Results

### Number of severe RSV cases and age distribution

**Figure 1:**
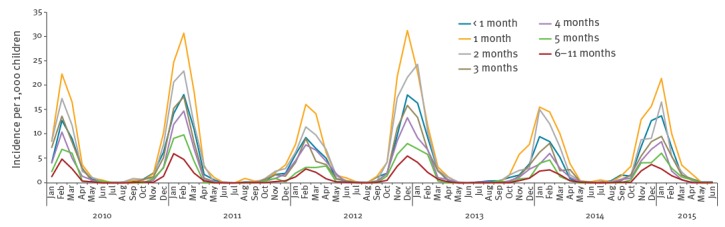
Monthly respiratory syncytial virus-hospitalisation incidence per 1,000 children up to 12 months of age, in Denmark, during the study period, 1 January 2010–26 June 2015

During the study period of 1 January 2010 to 26 June 2015, 10,843 hospitalisations had an ICD-10 code compatible with RSV infection. When linking the NPR information on hospital admission (Table 1) periods to the RSV test-positive results in MiBa by the unique personal identification number an additional 1,487 (12.1%) unique episodes of RSV-associated hospitalisations were identified in children with other respiratory diagnoses. In total, 12,330 RSV-associated hospitalisations were identified in the cohort of children < 5 years of age during the study period representing 346,444 person-years. The average incidence of severe RSV cases expressed as annual RSV-associated hospitalisation rate was 7.1 per 1,000 children < 5 years of age with 9,539 (77%) of the hospitalisations occurring among children < 12 months of age, and 7,409 (60%) in children < 6 months of age. The average annual hospitalisation rates were 45.9 per 1,000 children < 6 months of age and 29.4 per 1,000 children < 12 months of age, respectively, with the highest rate of 75.7 per 1,000 infants 1–2 months of age (Table 2). The monthly RSV-hospitalisation incidence per 1,000 children during the 5 year study period also consistently identified the infants 1 month of age as having the highest rate of hospitalisation (Figure 1).

**Table 2:** Annual incidence rates of respiratory syncytial virus associated hospitalisations per 1,000 persons by age group, in Denmark, in the study period, 1 January 2010–26 June 2015

Age in months	Cases	Person-years	Incidence rate (cases per time-at-risk)	95% confidence intervals
< 1	1,215	26,890	45.2	42.7–47.8
1	2,037	26,907	75.7	72.5–79.1
2	1,574	26,903	58.5	55.7–61.5
3	1,088	26,915	40.4	38.1–42.9
4	882	26,950	32.7	30.6–35.0
5	613	26,959	22.7	21.0–24.6
6-11	2,130	163,205	13.1	12.5–13.6
All 0–11	9,539	324,729	29.4	28.8–30.0
12–23	2,138	340,623	6.3	6.0–6.5
24–35	470	349,317	1.3	1.2–1.5
36–47	120	356,851	0.3	0.3–0.4
48–59	63	360,702	0.2	0.1–0.2

Based on the National Microbiology database and the National Patient Discharge Registry data.

On average, 2,466 Danish children < 5 years of age were hospitalised due to severe RSV infection annually, ranging from the lowest 1,456 in the 2013/14 season to the highest 2,834 in the 2012/13 season.

The median age for RSV hospitalisation was 7.7 months (interquartile range (IQR): 1.9–11.1 months).

### Seasonality

RSV-associated hospitalisations demonstrated a distinct seasonal variation with peaks in the winter months of December through March and almost no cases during the summer months of June through September.

Every other year the RSV season had an early start in weeks 46 to 48 (Figure 1), and was rather mild in terms of hospitalisations (2009/10, 2011/12, and 2013/14). Every alternating year the RSV-season would start a few weeks later (week 50–52) and be characterised with a markedly higher RSV-hospitalisation incidence (2010/11, 2012/13, and 2014/15).

### Sex distribution

Of the 12,330 hospitalisations, 6,901 (55.9%) occurred among young boys resulting in an overall male to female ratio of 1.3. The sex ratio (male to female) was highest in the first months of life, peaked among infants 4 months of age (1.4) (n = 882) and gradually levelled out with increasing age to 1.0 in children 4 years of age (n = 63).

### Mortality

During the study period, one child was recorded in the Register of Causes of Death registry as having died of RSV-associated infection. Additionally, by use of the indirect method, 28 children of the total of 12,330 RSV cases were registered as no longer alive in the CRS. When linking the date of death to the RSV-associated hospitalisation admission and discharge dates, four of the 28 children were identified as having died either during hospitalisation or within 30 days of being discharged from hospital with a RSV-specific diagnosis and/or RSV positive test result. Of the deaths identified by use of the indirect method two children died in the hospital whereby the discharge date and date of deaths are identical, one child died within 24 hours after being discharged and one child died 3 days after being discharged, respectively. In total, five deaths among 12,330 RSV cases < 5 years of age resulted in a case fatality rate of 0.04%. The median age of the RSV-associated deaths was 6.5 months (IQR: 1.0–9.8 months). Four of the five patients had underlying conditions (muscular dystrophy and myopathy, bronchopulmonary dysplasia, Steno Fallot’s tetralogy, and multiple congenital malformations), and two of these were born preterm < week 28, which might have predisposed them for severe outcome of RSV disease.

### Hospitalisation length of stay

**Figure 2:**
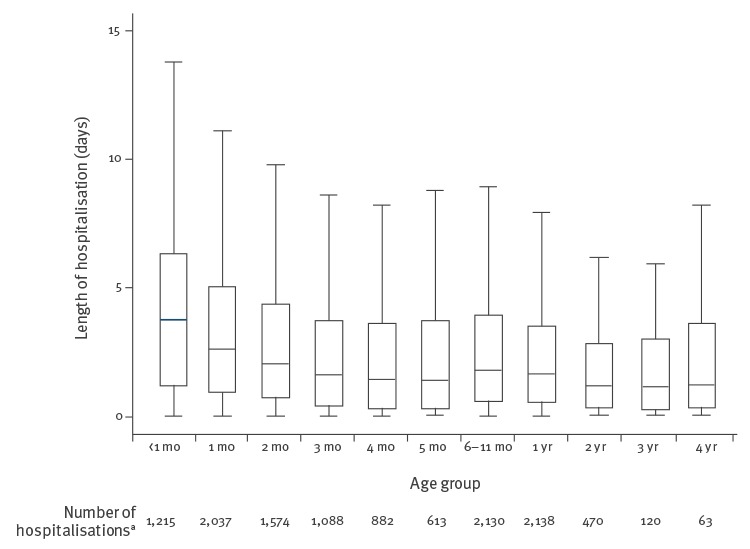
Duration in days of respiratory syncytial virus-associated hospitalisations among children < 5 years of age by age groups, in Denmark, during the study period, 1 January 2010–26 June 2015

^a^ Hospitalisations longer than 15 days (n = 100) were included in all calculations but do not figure inside the diagram for illustration purposes. Below the diagram however, on the line entitled ‘numbers of hospitalisations’, such hospitalisations are included.

The median length of stay in hospital for the entire cohort was 1.96 days (IQR: 0.67–4.17). It was higher among children younger than 3 months of age (median length-of-stay: 2.71 days; IQR: 0.92–5.21) compared with children 3 months or older (median length-of-stay: 1.62 days; IQR: 0.46–3.71), (p < 0.001) (Figure 2).

### Direct healthcare expenses

Of the 12,330 RSV associated admissions 90.2% lasted < 6 days (n = 11,123), and 9.8% (n = 1,207) lasted ≥ 6 days (range: 6–103 days) (Figure 2). The total direct medical costs of RSV-associated hospitalisations therefore amounted to ca EUR 4.1 million (ca DKK 30.5 million) per year.

## Discussion

A vaccine’s effectiveness to prevent severe disease, hospitalisations and deaths is of paramount importance when weighing pros and cons for introduction of new vaccines into national immunisation programmes. Due to under-registration, misclassification and lack of national guidelines for RSV-testing, hospital diagnosis statistics are rarely sufficient to estimate incidence or prevalence of RSV-associated disease. In the present study, we have investigated the burden of severe RSV-associated disease in children below 5 years of age in Denmark using a combination of population-based national registries. We demonstrate how combining nationwide hospitalisation data and microbiological test results, increases the estimates of RSV hospitalisations compared with estimates based on the national hospital discharge registry alone.

The incidence of severe RSV infections was highest among infants and young toddlers and peaked in the 1 to 2 month-old infants. This is in line with a study by Hall et al. 2013 [22], where the highest age-specific rate was observed in 1 month-old infants. This age specific distribution may reflect rapidly declining levels of protecting maternal antibodies after birth. This hypothesis is consistent with findings in a recent study from Turkey where anti-RSV IgG positivity in offspring dropped from 83% at birth to 73% at 1 month and 6% at 3 months of age [29]. As the incidence of RSV-hospitalisations was high already at 1 month of age, the vaccination strategy could be either vaccination of the infant shortly after birth or maternal vaccination before birth. Vaccination of the newborn is challenging due to the immaturity of the neonates’ immune system and the passively received maternal antibodies, which further can impair the response. Maternal vaccination before birth, boosting the titre of maternal antibodies [30], might provide a more effective protection of the neonates as compared with vaccination of a newborn.

Considering a universal RSV immunisation programme with an expected moderate to high vaccine-effectiveness against hospitalisation and deaths, a considerable fraction of RSV-associated hospitalisations likely could be avoided per year in Denmark. Whether a future RSV vaccine would be cost-saving is too early to predict as weighing the health benefits of vaccination against its costs requires a measure like quality-adjusted life years, which takes both reduced illness and death into account, and subsequent cost-effectiveness analyses in which the healthcare-associated as well as the societal costs are considered [31]. In this study, the cost estimates due to RSV were only based on hospitalisation-associated expenses and did not take into account indirect costs from socio-economic loss, or from absenteeism of parents of infants older than 6 months (as the granted Danish maternity leave is minimum 6 months). 

The observed seasonal variations in RSV hospitalisations with the highest incidence during the winter months from December through March were in line with findings in northern Europe [32-34] and in temperate climate zones of the United States [35,36]. The data suggested that fewer hospitalisations occurred in years with an early start of the RSV season as compared with years with a later start of RSV seasonal activity. The 2-year cyclic pattern observed in Denmark for RSV seasons is in line with other country reports from e.g. Finland and Switzerland [32,33,37-40]. 

In the RSV-affected children under 5 years, the median length of hospitalisation was 1.9 days, and not surprisingly longer (2.7 days) in infants < 3 months of age. Other studies have reported similar age-associated differences in length of stays among RSV-hospitalised infants of 2 days [41], 3 days [42-44], 4 days [45,46],  and 6 days [47]. Hon et al [48] differentiate length of stay further with 8.5 days for paediatric ICU patients and 3 days for children not in the ICU [48]. As many studies only provide the mean length of stay, despite the observations rarely being normally distributed, this challenges our ability to identify and compare our findings to that of relevant compatible study settings.

We examined deaths in the Register of Causes of Death and deaths occurring during or within 30 days of an RSV-associated hospitalisation as possibly due to RSV. The majority of the children had underlying illness during their last hospitalisation which likely increased their risk of a fatal outcome. Therefore the numbers may be overestimated. On the other hand, some children may have died of RSV without being hospitalised or in the aftermath of an RSV hospitalisation due to complications of the infection. In the present study, a total of five of 12,330 cases died within 30 days of hospital admission. Compared with a case definition based on RSV specific ICD-10 codes alone, which yielded one fatal case, combining RSV-associated ICD-10 code with a laboratory-confirmed RSV-test result captured four more children. Four of the five children had known comorbidities. The low mortality rate of 0.04% due to RSV infection in our study is in accordance with findings in other western countries e. g. in Spain, where a 15-year study found a case fatality rate of 0.14% in the same age group [49,50].

Limitations and strengths: Data on comorbidity were only included for analyses of deceased children and not on all cases included. Also, although the NPR contains ICU data, these were not taken into consideration in this work. A major strength of the study however, is access to national hospitalisation data and a real-time national microbiology database where linkage between registries using unique personal identifier enables validation of the use of national patient discharge registries in assessment of burden of severe RSV-disease. Further, the uniformity of data collected in the microbiology database including test negative results ensures a strong platform for national disease burden estimates. Our validation of the national patient discharge registry using national RSV laboratory test data demonstrates, not surprisingly, that analyses of RSV-hospitalisation burden based on ICD-10 codes alone result in underestimation of the true burden.

In conclusion, our study provides population based estimates on the incidence and costs of RSV-associated hospitalisations in children under 5 years of age in a western country. An effective RSV vaccine could reduce costs and burden related to RSV hospitalisations considerably. We document how severe RSV infection predominantly affects the youngest children and, demonstrate that the most vulnerable age group includes the infants 1 to 2 months of age. Knowledge of the age-specific RSV burden in infants is crucial for future decisions on vaccination strategies including the choice of using maternal and/or infant vaccination.
